# Z-DNA as a Tool for Nuclease-Free DNA Methyltransferase Assay

**DOI:** 10.3390/ijms222111990

**Published:** 2021-11-05

**Authors:** Sook Ho Kim, Hae Jun Jung, Seok-Cheol Hong

**Affiliations:** 1 Center for Molecular Spectroscopy and Dynamics, Institute for Basic Science, Seoul 02841, Korea; larckim83@gmail.com (S.H.K.); zoring@korea.ac.kr (H.J.J.); 2 Department of Physics, Korea University, Seoul 02841, Korea; 3 GRI-TPC International Research Center, Sejong University, Seoul 05006, Korea

**Keywords:** Z-DNA, DNA methylation, nuclease-free, single-molecule FRET, methylcytosine sensitive, natural DNA methyltransferase inhibitors

## Abstract

Methylcytosines in mammalian genomes are the main epigenetic molecular codes that switch off the repertoire of genes in cell-type and cell-stage dependent manners. DNA methyltransferases (DMT) are dedicated to managing the status of cytosine methylation. DNA methylation is not only critical in normal development, but it is also implicated in cancers, degeneration, and senescence. Thus, the chemicals to control DMT have been suggested as anticancer drugs by reprogramming the gene expression profile in malignant cells. Here, we report a new optical technique to characterize the activity of DMT and the effect of inhibitors, utilizing the methylation-sensitive B-Z transition of DNA without bisulfite conversion, methylation-sensing proteins, and polymerase chain reaction amplification. With the high sensitivity of single-molecule FRET, this method detects the event of DNA methylation in a single DNA molecule and circumvents the need for amplification steps, permitting direct interpretation. This method also responds to hemi-methylated DNA. Dispensing with methylation-sensitive nucleases, this method preserves the molecular integrity and methylation state of target molecules. Sparing methylation-sensing nucleases and antibodies helps to avoid errors introduced by the antibody’s incomplete specificity or variable activity of nucleases. With this new method, we demonstrated the inhibitory effect of several natural bio-active compounds on DMT. All taken together, our method offers quantitative assays for DMT and DMT-related anticancer drugs.

## 1. Introduction

DNA methylation is one of the essential epigenetic modifications in the genome, playing critical roles in development, differentiation, and tumorigenesis [1,2,3,4,5,6,7]. Promoter hypermethylation of tumor suppressor genes is commonly observed in cancers [8,9,10,11]. Thus, DNA methylation is an important bio-marker for cancers [7,9] and the detection of DNA methylation is significant in epigenetic analysis and cancer biology [3,4,8,10,12]. In eukaryotes, DNA methylation, more specifically cytosine methylation, is enzymatically induced by DNA methyltransferases (DMT), which transfer the methyl group from the cofactor, S-adenosyl-L-methionine (SAM or adoMet), to the C5 position of cytosine within a cytosine–guanine (CpG) dinucleotide [13,14,15]. DNA methylation, reversible as an epigenetic modification and responsible for the fate of a cell [4,16,17], emerges as a promising therapeutic target in cancer treatment [5,18,19]. There has been a great deal of effort to develop effective drugs to interfere with the action of DMT, and, thus, to regulate the state of DNA methylation in the genome as well as on a specific gene [3,5,18,19,20,21].

To probe DNA methylation and characterize its profile, a number of methods have been developed to detect DNA methylation [12,21,22,23,24,25,26,27,28,29,30,31]. To determine which part of the genome is methylated, the bisulfite genomic sequencing method is widely used [9,22,25,30]. The methylation-specific polymerase chain reaction (PCR) technique further improved the sensitivity and signal strength by PCR with primers specific for methylated substrates [23].

A vast majority of studies for DNA methylation rely on CpG methylation-sensitive restriction enzymes [21,24,26,27,28,29,31,32,33,34,35]. Recognition of methylcytosine at a CpG site by such nucleases is the first step in a broad range of DMT assays. In this way, one can check whether a certain locus is methylated, and detect the activity of DMT on a well-defined (cognate) sequence. One can also test the efficacy of DMT inhibitors by measuring the probability of DNA methylation in their presence. Since these assays critically rely on the activity of nucleases, quantitative evaluation of the activity of DMT might be limited by variable nuclease activity. The assays based on restriction enzymes are not sensitive to DNA hemi-methylation because the cleavage efficiency by methylation-dependent nucleases such as DpnI is compromised for hemi-methylated DNA substrate [36]. Methylated cytosines (methylcytosines) can be recognized alternatively by methyl-specific antibodies [31,37,38], but the antibody-based methods are dependent upon the sensitivity and specificity of antibodies.

Many of the methods developed for DMT assays utilize a variety of amplification strategies including nanoparticle binding, metal clustering, multi-turnover enzymatic amplification, or product accumulation to amplify feeble signals directly from oligonucleotides [23,24,29,31,32,33,34,35,37,39,40,41,42]. Amplification-based methods are lauded because the step(s) of amplification can tremendously boost the level of the signal and permit signal detection by common commercial detectors or even bare eyes [40]. Perhaps for most diagnostic tests, amplification-based assays are useful and good enough. For quantitative analysis, such assays should be applied with caution because final results are yielded after a cascade of (amplification) steps and have no memory of the initial status of DNA methylation. The level of the final signal depends on various factors such as detection errors and the concentration and quality of reagents, which contribute to measurement uncertainty. Moreover, the preparation of special nanoparticles, enzymes, and a battery of reagents, often required for such assays, add complexity to these methods. Several single-molecule approaches such as solid-state nanopore technology and single-molecule fluorescence microscopy have been applied and have exhibited the fast and precise detection of methylcytosine in a CpG site with antibodies or methyl-binding proteins [38,43,44,45].

Here, we report a new method to characterize the activity of DMT and the effect of inhibitor drugs, utilizing a methylation-sensitive conformational transition of DNA. The transition from B-DNA to Z-DNA (B-Z transition) occurs efficiently in a sequence of CpG dinucleotide repeat, and the B-Z transition occurs even more efficiently in the same sequence with the cytosines methylated [46,47] because the methyl moieties attached to the cytosines stabilize the Z-DNA conformation by excluding water from the hydrophobic pocket that exists in the conformation [48]. DNA methylation can be measured quantitatively by measuring the populations of B- and Z-state. In our previous work, we demonstrated that the single-molecule FRET (smFRET) experiment is an ideal technique to detect the B-Z transition and the methylated CpG repeat undergoes the transition at lower monovalent (Na^+^) or divalent (Mg^2+^) cation concentrations than the non-methylated, otherwise identical sequence [49]. Like other non-B-DNA conformations [50,51], Z-DNA can make a functional nano-device due to its intriguing physical and chemical properties.

In this work, we established the quantitative dependence of Z-DNA formation on the degree of cytosine methylation, the sensitivity of which is the logic behind this B-Z transition-based DMT assay. In contrast to the aforementioned assays for a single CpG site (just all or none type assay), this method can detect the spectrum of cytosine methylation because the probability of the Z-state changes continuously with the degree of cytosine methylation.

Our smFRET-based method developed here can test the action of DMT and screen DMT inhibitor drugs in quantitative manners. This method can distinguish different degrees of cytosine methylation in a single DNA molecule. The high sensitivity of the technique makes it possible to reduce the amount of sample necessary for each assay and circumvent the amplification steps such as PCR. Moreover, target molecules maintain their molecular integrity and methylation state without being cleaved. The non-destructive and amplification-free approach circumvents the risk of introducing artefactual effects augmentable by a cascade of subsequent reactions and consequently of undermining reliable quantification. One key advantage of our method is to spare methylation-specific nucleases, exempt from the drawbacks caused by nucleases. Furthermore, we measured and confirmed the inhibitory effect of several dietary nutraceutical compounds on DMT activity. The technique would certainly find excellent applications with distinct benefits unavailable so far in other traditional approaches.

## 2. Results and Discussion

### 2.1. The Population of the Z-State in CG Repeat Sequences Is Dependent on the Degree of DNA Methylation

**Figure 1:**
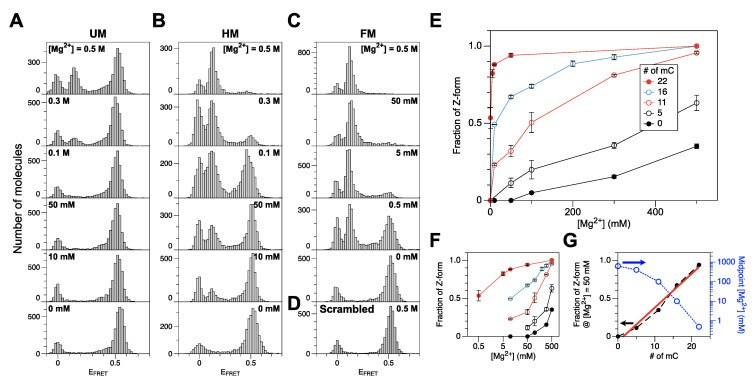
Populations of the Z-DNA state in the DNA cores with different degrees of cytosine methylation under various salt conditions. (**A**) FRET efficiency histograms for a CG core with no methylation (UM) (bottom to top: no Mg^2+^ (100 mM Na^+^ only), 10 mM, 50 mM, 100 mM, 300 mM, 500 mM Mg^2+^). (**B**) FRET efficiency histograms for a CG core with hemimethylation (HM) (bottom to top: no Mg^2+^ (50 mM Na^+^ only), 10 mM, 50 mM, 100 mM, 300 mM, 500 mM Mg^2+^). (**C**) FRET efficiency histograms for a CG core with full methylation (FM) (bottom to top: no Mg^2+^ (100 mM Na^+^ only), 0.5 mM, 5 mM, 50 mM, 500 mM Mg^2+^). (**D**) FRET efficiency histogram for a scrambled Core with 500 mM Mg^2+^. (**E**) Fraction of Z-form vs. [Mg^2+^] for DNA molecules with various degrees of cytosine methylation. (**F**) Panel (**E**) redrawn with [Mg^2+^] in log scale. (**G**) Fraction of Z-form at [Mg^2+^] = 50 mM and midpoint [Mg^2+^] for cores with various degrees of cytosine methylation. Dashed lines connect data points. The Z-DNA populations increase linearly with the degree of cytosine methylation as revealed by the well-correlated linear fits (red solid line). The midpoint Mg^2+^ concentrations for various cores (blue dotted) are shown in log scale (shown on the blue right *y*-axis). FRET efficiency histograms for QM and TM cores are shown in Supplementary Materials, Figure S3.

To check whether DNA methylation promotes Z-DNA formation, we measured FRET efficiencies from unmethylated (UM), hemi-methylated (HM), and full-methylated (FM) CG repeat sequences as well as other CG repeat sequences (QM, TM: see Section 3.1 and Table 1) with intermediate degrees of methylation, all of which contained a FRET dye pair separated by 14 bp. The UM core underwent the B-Z transition at very high [Mg^2+^]. Figure 1A shows that the B-Z transition for the UM core DNA required [Mg^2+^] higher than ~0.5 M (the midpoint value is known to be 0.7 M). A peak at E_FRET_ ~0 in the histogram corresponds to donor-only DNA molecules (with the acceptor dye missing or photobleached). Donor-only molecules exist in other histograms as well. The HM core underwent the B-Z transition at lower [Mg^2+^] with the midpoint [Mg^2+^] near 100 mM, nearly one order of magnitude less than the value for the UM core (Figure 1B). Above [Mg^2+^] ~300 mM, the HM core mainly exists in Z-form. An HM DNA molecule can be also prepared enzymatically (Supplementary Materials, Figure S2A, and the Supplementary Materials Text), and the ‘enzymatic HM core’ also exhibits a similar trend of transition but with less efficiency because of the imperfect methylation of the CG core presumably due to interference by labeled dyes. The enzymatic HM DNA can be an option although the sample preparation is rather complicated, and the enzymatic HM core behaves rather like the QM than the HM core (Supplementary Materials, Figure S2B).

**Figure 2:**
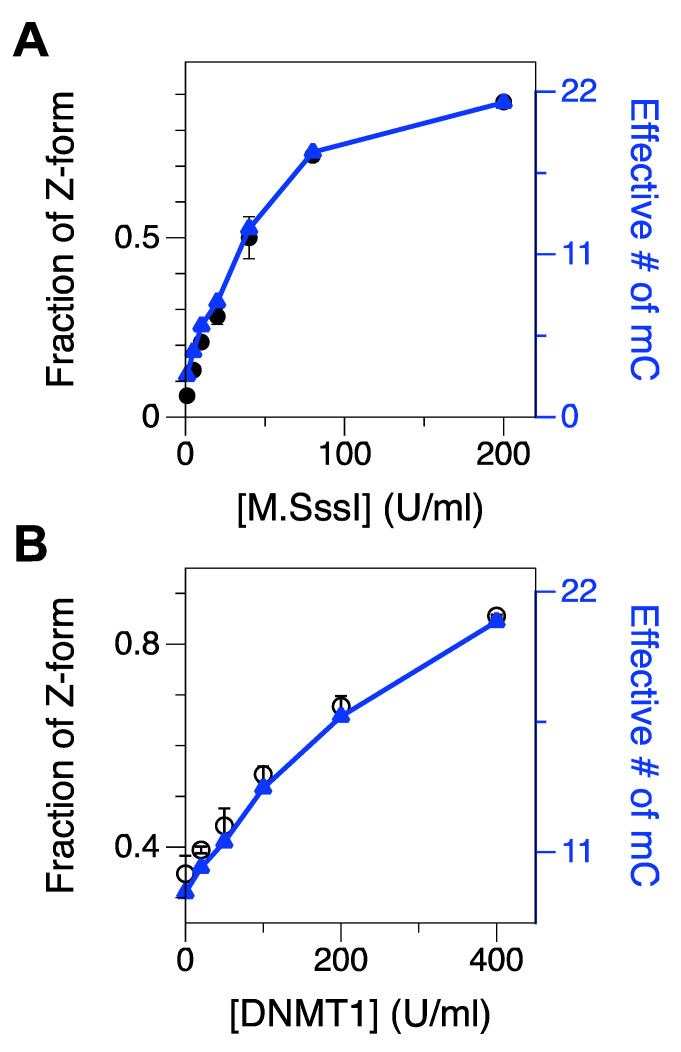
Fractions of Z-form of an initially UM or HM CG core after 2-h incubation with various concentrations of M.SssI (1–200 U/mL) or DNMT1 (20–400 U/mL), respectively. The populations were measured in the presence of 50 mM Mg^2+^. FRET efficiency histograms for cores treated with various concentrations of DMT are shown in Supplementary Materials, Figure S5. (**A**) Fraction of Z-form vs. [M.SssI] (black) overlaid with the effective number of methylated cytosines (blue). (**B**) Fraction of Z-form vs. [DNMT1] (black) overlaid with the effective number of methylated cytosines (blue).

The FM core underwent the B-Z transition with the midpoint [Mg^2+^] of ~0.5 mM (Figure 1C), which is within the range of physiological [Mg^2+^] [52]. This midpoint concentration is about two orders of magnitude less than the value for HM core. At higher concentrations (5 and 50 mM), the FM core mainly exists in Z-form. An FM DNA molecule can also be prepared enzymatically, and the ‘enzymatic FM core’ also exhibits a similar behavior but with less efficiency (enzymatically methylated by 200 U/mL of M.SssI (Figure 2A) as in Ref. [49] and 400 U/mL of DNMT1 (Figure 2B), respectively) again because of the imperfect methylation of the CG core. Since high [Mg^2+^] has no effect on the FRET efficiency from a scrambled sequence (Figure 1D), the FRET change is sequence-specific and originates from the B-Z transition in CG repeat sequences.

The QM (‘5–0’) core exhibited a transitional behavior between the UM and HM cores (Figure 1E and Supplementary Materials, Figure S3A). As expected, the TM (‘5–11’) core exhibited intermediary behavior between the HM and FM cores (Figure 1E and Supplementary Materials, Figure S3B).

Relative Z-DNA populations of CG cores with various degrees of cytosine methylation are shown in Figure 1E,F as a function of [Mg^2+^]. We found that the requirement for Mg^2+^ in the B-Z transition decreases dramatically with an increasing number of methylcytosines (Figure 1G (blue, open)). We could maximize the *resolution* of the number of methylated cytosines simply by choosing different [Mg^2+^] for different ranges of cytosine methylation. At [Mg^2+^] = 500 mM, UM, QM, and HM cores are clearly distinguished while FM, TM, and HM are well-resolved at [Mg^2+^] = 10 mM (Figure 1F). From these data, we extracted an empirical relationship between the number of methylcytosines (or fraction of methylcytosines) and the probability of the Z-state at [Mg^2+^] = 50 mM, which is almost linear (Figure 1G (black dot; red, solid line: fit)). This makes a useful reference to estimate the degree of DNA methylation from a relative Z-DNA population measured by smFRET assays.

### 2.2. Methylation of CG Cores with M.SssI and DNMT1

M.SssI is a commercially available CpG DMT [53]. It is capable of inducing de novo methylation from unmethylated to full methylated DNA. It is a more potent and active enzyme than DNMT1, another target enzyme studied in this work. Moreover, M.SssI is used to screen DNMT1 inhibitors as an alternative to DNMT1 as they are structurally analogous enzymes [54,55,56] and many DNMT1 inhibitors are shown to inhibit M.SssI and vice versa [57,58,59]. It is, however, ultimately important to study DNMT1 because it is biologically significant as it is directly related to human epigenetic functions.

We started with unmethylated DNA (UM core) to test the methylation activity of M.SssI. We stopped the reaction of DNA methylation by heat inactivation at different time points (0~4 h) after the onset of the reaction (Supplementary Materials, Figure S4A,B). We found that the enzyme activity leveled off after 30-min incubation.

We tested the effect of M.SssI concentration on methylation as shown in Figure 2A and Supplementary Materials, Figure S5A. In that assay, we stopped the reaction of DNA methylation after 2-h incubation. As expected, the population of Z-DNA, indicative of the extent of cytosine methylation, increased consistently with the concentration of M.SssI, and beyond the concentration of ~100 U/mL, the population of Z-DNA gradually leveled off. Using the relationship of the number of methylcytosines and the population of the Z-state acquired above, we were able to estimate the effective number of methylcytosines in the core. At the maximum resolution for methylated cytosines (1~2 m^5^C from Figure 1F), the detection limit for DMT would be ~5–10 U/mL in our assay (Figure 2A and Supplementary Materials, Figure S5A), which is similar to the value reported in Ref. [34]. Assays optimally detecting single methylated cytosines with nucleases or antibodies, however, exhibit remarkably lower detection limits for DMT [38,60,61]. With [M.SssI] = 200 U/mL, M.SssI methylates almost all the cytosines in the core sequence except for cytosines near the dye molecules.

Similarly, we also tested the methylation activity of DNMT1 in a concentration-dependent manner [62] (Figure 2B and Supplementary Materials, Figure S5B). As DNMT1 is known to act on hemi-methylated CG repeat sequences, we used the hemi-methylated CG core (HM) as a substrate. The midpoint [Mg^2+^] for the HM core was approximately 100 mM and at [Mg^2+^] = 50 mM, ~39% of molecules were in Z-state and the rest in B-state. Then, we incubated the HM core with DNMT1 at various concentrations (0, 20, 50, 100, 200, and 400 U/mL) to test whether DNMT1 could methylate the non-methylated strand (CG2) in the HM core. Then, we found that the population of the Z-state after DNMT1 treatment was significantly larger than that of the HM core (as a no-DNMT1 control because the DNA substrate for this assay was hemi-methylated) and, the population of the Z-state, especially for data with [DNMT1] = 400 U/mL, is comparable to that of FM DNA (Figure 2B and Supplementary Materials, Figure S5B), which supports that DNMT1 can almost fully methylate the HM DNA substrate.

To test de novo methylation of DNA by DNMT1, we also asked how well DNMT1 could methylate unmethylated DNA (UM). As expected, the de novo methylation by DNMT1 (100 and 200 U/m) is inefficient (Supplementary Materials, Figure S6), consistent with the known inefficiency of DNMT1 on unmethylated DNA [24]. This supports its in vivo role as a DMT working for the replication and inheritance of epigenetic patterns in semi-conservative DNA replication.

### 2.3. Natural Dietary Compounds Effectively Suppress the Methylation Activity of Both M.SssI and DNMT1

We tested the effect of natural compounds known as DNMT1 inhibitors on the methylation activity of DMT. We added different inhibitor compounds at various concentrations to the reaction mixture. We prepared *EGCG* (*epigallocatechin gallate*), polyphenol naturally found in green tea leaves, in deionized water [57,63,64], and *curcumin* from turmeric (rhizomes of *Curcuma longa*), medicinal ingredient of an Indian condiment, curry, in 2.5% DMSO in water. Curcumin’s pharmacological effect has been documented as a DNMT1 inhibitor over years [21,28,35,65,66,67,68,69,70,71]. Genistein, isoflavone naturally found in soybeans and coffee beans, was also prepared in ~1.75% DMSO in water and tested as a DMT inhibitor [21,28,35,39,72,73].

**Figure 3:**
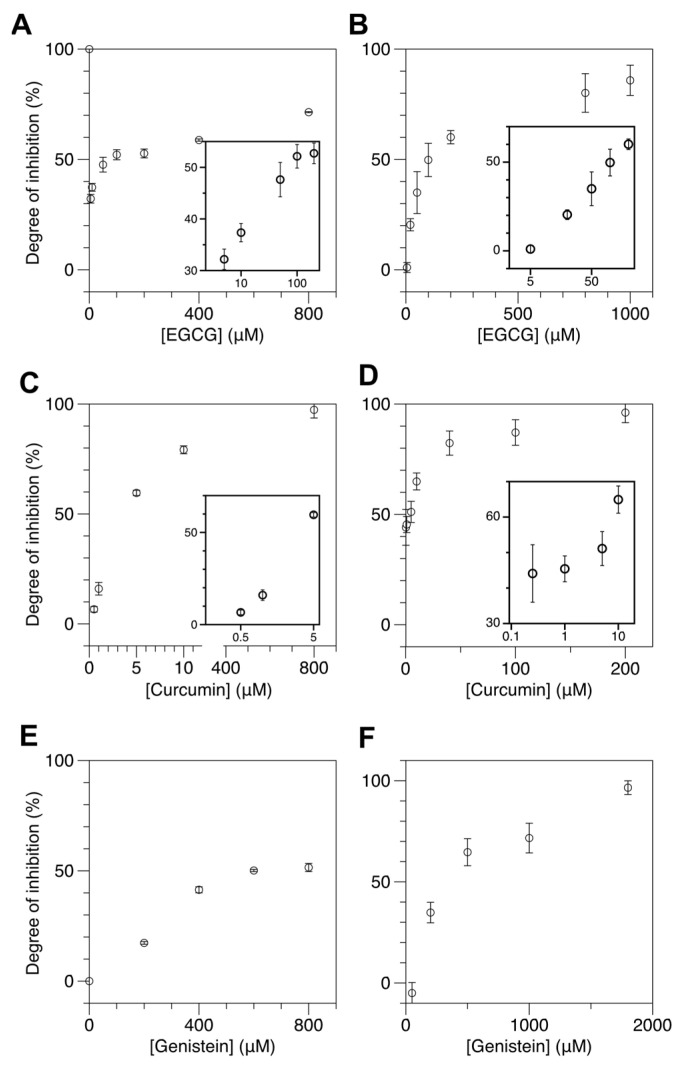
Natural dietary compounds suppress cytosine methylation in cores efficiently as revealed by reduced Z-DNA formation in the samples treated with those compounds. The concentration of SAM used was 160 μM. FRET efficiency histograms for cores incubated with DMT in the presence of various kinds and concentrations of natural inhibitors are shown in Supplementary Materials, Figure S7. (**A**,**B**) DOI of DMT activity by EGCG at various concentrations ((**A**) 5, 10, 50, 100, 200, 400, 800 μM for M.SssI and (**B**) 5, 20, 50, 100, 200, 800, 1000 μM for DNMT1). (**C**,**D**) DOI of DMT activity by curcumin at various concentrations ((**C**) 0.5, 1, 5, 10, 800 μM for M.SssI and (**D**) 0.25, 1, 5, 10, 40, 100, 200 μM for DNMT1). (**E**,**F**) DOI of DMT activity by genistein at various concentrations ((**E**) 200, 400, 600, 800 μM for M.SssI and (**F**) 50, 200, 500, 1000, 1800 μM for DNMT1). For EGCG and curcumin, the DOI values for low inhibitor concentrations are presented in insets with the inhibitor concentration in log scale.

Figure 3A,B shows the degree of inhibition (DOI) for M.SssI and DNMT1 as a function of EGCG concentration and that the degree of inhibition increases in a concentration-dependent manner. Here we define the degree of inhibition as:

$$\mathrm{DOI}=\frac{P\left(Z;\mathrm{DMT}\right)-P\left(Z;\mathrm{DMT}+\mathrm{drug}\right)}{P\left(Z;\mathrm{DMT}\right)-P\left(Z;0\right)}$$

where P(Z;0) is the population of Z-DNA without any treatment (neither DMT nor inhibitor drug), P(Z;DMT) the Z-DNA population after incubation with DMT, and P(Z;DMT+drug) the Z-DNA population after incubation with DMT together with an inhibitor drug. Therefore, if the drug has no effect, and, thus, P(Z;DMT+drug) = P(Z;DMT), it would be the case of zero inhibition (DOI = 0). On the opposite limit, the inhibition would be complete (DOI = 1) if P(Z;DMT+drug) completely reduces to the level of P(Z;0). We employed the UM and HM molecules as substrates for M.SssI and DNMT1, respectively. We used 50 mM Mg^2+^ and applied 80 U/mL M.SssI or 100 U/mL DNMT1. As shown in Figure 3A, the IC_50_ value of EGCG was approximately 80 μM for M.SssI. For DNMT1, the IC_50_ value of EGCG was approximately 100 μM. These values are somewhat larger than the IC_50_ values reported in a cell-based assay [63] and significantly larger than the IC_50_ values reported in an in vitro assay [57]. While the discrepancy in IC_50_ between our method and cell-based assays is not surprising due to their markedly different experimental settings, the discrepancy in IC_50_ between our method and the in vitro assay needs to be accounted for. The main source for the discrepancy is the difference in the concentration of SAM used in each assay. To reduce the reaction time, we used approximately 10 times more SAM (~160 μM) than used in other molecule-based studies (~10–20 μM). Therefore, we needed more inhibitors to reduce the reaction rate. To check this possibility, we reduced SAM to 10 μM and repeated the inhibitor assay. As shown in the Supplementary Materials, Figure S8, the new IC_50_ value of EGCG was approximately 5 μM or less, which is comparable to the reported values [57]. Thus, the large IC_50_ value from our assay was simply due to the concentration difference of SAM, not due to a difference in sensitivity.

Next, we tested the effects of curcumin and genistein on DNA methylation by DMT (Figure 3C–F). These drugs are available as over-the-counter food supplements. Figure 3C,D shows the DOI for M.SssI and DNMT1 as a function of curcumin concentration and that the DOI increases in a concentration-dependent manner. Again, we used the UM and HM molecules as substrates for M.SssI and DNMT1, respectively, and used the same concentrations of [M.SssI], [DNMT1], and [Mg^2+^] as in EGCG assays. As shown in Figure 3C, the IC_50_ of curcumin is approximately 3.6 μM for M.SssI. For DNMT1, the IC_50_ of curcumin is approximately 1.3 μM. These values are considerably larger than the IC_50_ values reported from various molecule-based studies [68]. Again, the discrepancy should be due to the concentration difference of SAM, not the technical limitation of our method.

Figure 3E,F shows the DOI for M.SssI and DNMT1 as a function of genistein concentration and that the DOI increases in a concentration-dependent manner as expected. We used the same experimental conditions as in the assays for the other drugs. As shown in Figure 3E, the IC_50_ of genistein is approximately 600 μM for M.SssI. For DNMT1, the IC_50_ of genistein is approximately 400 μM. To our knowledge, the inhibitory effect of genistein on M.SssI has not been investigated.

Our DNA methylation assays with these drugs and M.SssI show that those DNMT1 inhibitors also have an inhibitory effect on M.SssI, suggesting that putative DNMT1 drugs can be also tested to M.SssI likely because the molecular mechanisms by M.SssI and DNMT1 have considerable similarity and can be interfered with by those drugs. These DMT inhibitors display dissimilar inhibition efficacies. Curcumin is the most effective inhibitor among the three with the lowest IC_50_ value (~a few μM) for DNMT1 and M.SssI. EGCG is the second most effective inhibitor with an IC_50_ value of approximately a few tens of μM. Genistein is the least effective of all with an IC_50_ value of approximately several hundred μM. It appears that curcumin and EGCG act on the two DMT enzymes somewhat differently at low concentrations, the former acting marginally more effectively on DNMT1 while the latter on M.SssI. Such an issue shall be dealt with in future studies.

## 3. Materials and Methods

### 3.1. Preparation of DNA Samples, DNA Methyltransferases, and DMT Inhibitors

The DNA oligonucleotides used in this study (dye-labeled or unlabeled; cytosine-methylated or unmethylated; see Table 1) were purchased from IDT, Inc. (Integrated DNA Technologies, Inc., Coralville, IA, USA). The sequence information of the oligonucleotides (CG1, CG2, m^5^CG1, m^11^CG1, and m^11^CG2) used in this study is shown in the Supplementary Materials, Table S1. One oligonucleotide (CG2) has biotin at its 3′ end for immobilization on a NeutrAvidin-coated glass substrate. Our tether molecule was prepared by hybridizing two complementary oligonucleotides (approximately 1 μM each) in T50 buffer (10 mM Tris, and 50 mM NaCl, pH 8.0) according to the standard procedure [74].

**Table 1:** DNA oligonucleotides used in this study and types of DNA cores and their compositions.

DNA Oligonucleotides	DNA Cores	Core Composition
CG1(Cy5)	UM (0-0)	CG1-CG2
CG2(biotin, Cy3)	QM (5-0)	m^5^CG1-CG2
m^5^CG1(Cy5)	HM (11-0)	m^11^CG1-CG2
m^11^CG1(Cy5)	TM (5-11)	m^5^CG1-m^11^CG2
m^11^CG2(Cy3)	FM (11-11)	m^11^CG1-m^11^CG2

DNA duplexes with different degrees of methylation were prepared in a combinatorial manner. For the unmethylated core (UM), two non-methylated strands (CG1 and CG2) were hybridized; for the hemi-methylated core (HM), an all-methylated strand (m^11^CG1) and a non-methylated strand (CG2) were hybridized; for the full-methylated core (FM), two all-methylated strands (m^11^CG1 and m^11^CG2) were hybridized. To have a less methylated molecule than HM, we hybridized a half-methylated strand (a CG1-type strand in which every other cytosine (total of five) is methylated (m^5^CG1)) and the unmethylated strand (CG2), acquiring a core molecule with five cytosines on one strand methylated (quarter methylation (QM), 5-0). To have a molecule in which the degree of methylation is between hemi- and full-methylation (three-quarter methylation (TM), 5-11), we hybridized the half-methylated strand (m^5^CG1) and the all-methylated strand (m^11^CG2). In contrast to CG2, m^11^CG2 does not have biotin at the 3′ end so TM and FM molecules were ligated to a short linker prepared by PCR with biotinylated dUTP (biotin-16-dUTP, Roche, Basel, Switzerland) for surface immobilization.

We also prepared methylated DNA molecules (with less-than-intended degrees of methylation) in an enzymatic way (see the Supplementary Materials). By comparing the enzymatically prepared molecules with the purchased synthetic ones for the B-Z transition, we could estimate the level of (incomplete) methylation in enzymatically methylated molecules.

We purchased both M.SssI (DNA methylase from *Spiroplasma* sp. strain MQ1) and human DNA methyltransferase 1 (DNMT1) from NEB (New England Biolabs, Inc., Ipswich, MA, USA). S-Adenosyl methionine (SAM or AdoMet) is supplied together with each purchase of M.SssI or DNMT1 (32 mM, 0.5 mL). We also purchased natural DMT inhibitors, curcumin, EGCG (epigallocatechin gallate), and genistein (Sigma-Aldrich, St. Louis, MO, USA).

### 3.2. DNA Methylation Reactions

**Figure 4:**
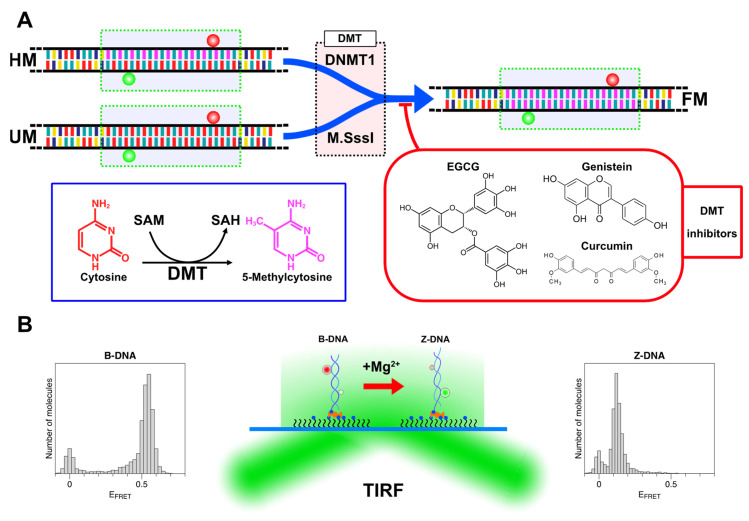
(**A**) Sample designs and main reactions of this work. DNA tether molecules with a 22-bp CpG repeat (green dotted box) with various degrees of cytosine methylation (bases are color-coded; A: yellow; T: indigo; G: teal; C: red; and mC (methylcytosine): purple) were prepared by hybridizing two dye-labeled complementary oligonucleotides (Cy3 (green ball) and Cy5 (red ball) on each oligonucleotide; FRET dye pairs were separated by 14 base pairs; See Supplementary Materials, Table S1, for sequence information) with a specific number of methylcytosines. Hemi-methylated (HM) and unmethylated (UM) molecules were the substrates for, and converted to, full methylated molecules (FM) by DNMT1 and M.SssI, respectively. DNA molecules were treated with DNA methyltransferases (DMT: DNMT1 or M.SssI) in a test tube. DMT enzymes changed cytosines (red) to methylcytosines (purple) by converting SAM to SAH and increased the degree of cytosine methylation in the DNA substrates as shown in the blue box. The reaction mixture was supplemented with one of the natural DMT inhibitors (EGCG, curcumin, or genistein) as shown in the red box. (**B**) Experimental setup and single-molecule detection strategy. Dye-labeled DNA tethers immobilized in the sample chamber are imaged in a TIRF microscope to measure FRET efficiency. The B-Z transition occurring to methylated DNA can be detected via a change in FRET efficiency in Mg^2+^-rich solutions. Representative FRET efficiency histograms for B-DNA (with low [Mg^2+^]; E_FRET_~0.5) and Z-DNA (with high [Mg^2+^]; E_FRET_~0.15) are shown as well. HM, UM, and QM tethers were immobilized on a glass substrate with biotin (blue ball) labeled at the 3′ end of the Cy3-labeled oligonucleotide while TM and FM tethers were immobilized via a biotinylated PCR fragment, which was ligated to the overhang (GATC) from the Cy5 labeled oligonucleotide (CG1 or methylated CG1) of TM or FM.

We performed the DNA methylation assay with DMTs (either M.SssI or DNMT1) in the absence or presence of one DMT inhibitor (EGCG, curcumin, or genistein) as shown in Figure 4A. DMTs can change cytosine to methylcytosine by converting SAM to SAH (S-adenosyl homocysteine). In the inhibitor-free assay, we added reagents to water (or 0, 1.75, and 2.5% DMSO in water in control reactions for inhibitor assays) in the following order: SAM, 10× buffer, DNA, and M.SssI or DNMT1. In the DNMT1 assay, the reaction mixture of 20 μL contained 25 nM DNA duplex and 20~400 U/mL (in the inhibitor-free assays) or 100 U/mL (in the inhibitor assays) DNMT1 in the reaction buffer (160 μM SAM and 100 μg/mL BSA in 1× DNMT1 buffer (200 mM NaCl, 50 mM Tris-HCl, 1 mM EDTA, 1 mM DTT, 50% glycerol, pH 7.5)). In the M.SssI assay, the reaction mixture of 20 μL contained 25 nM DNA duplex and 10~200 U/mL (in the inhibitor-free assays) or 80 U/mL (in the inhibitor assays) M.SssI in the reaction buffer (160 μM SAM in 1× NEB buffer 2 (50 mM NaCl, 10 mM Tris-HCl, 10 mM MgCl_2_, 1 mM DTT, pH 7.9)). The effect of DMSO itself was tested because it was used in a solvent for DMT inhibitors. We varied the concentration of DMSO solution up to 10% and found that the concentration below 5% had a negligible effect on DMT activity (see the Supplementary Materials, Figure S1; in contrast to Ref. [75], DMSO over 10% can suppress the enzyme activity). The mixture was incubated at 37 °C. After various lengths of incubation time (typically 2 h), the methylation reaction was stopped by heat inactivation of the enzymes at 65 °C for 20 min and the reaction mixtures were stored at −20 °C until the future experiments with the smFRET technique.

In the DMT inhibition assays, DMT inhibitors at various concentrations were added together with either M.SssI or DNMT1 when the enzyme was added to the reaction tube (Figure 4A). The reaction and analysis were performed identically except for the addition of inhibitors. In assays with EGCG, genistein, and curcumin (and corresponding control assays), the reaction mixtures were prepared in 0, 1.75, and 2.5% DMSO in water, respectively. In these assays, we varied the concentrations of the reagents (proteins or inhibitors) or incubation times.

### 3.3. Single-Molecule FRET-Based DNA Methylation Assay

The single-molecule FRET technique has been reviewed in numerous papers [76,77,78] and the setup used in our assay was also described previously (we used 532 nm CW laser (Coherent, Inc., Santa Clara, CA, USA), the power of which was ~3 mW at the laser. The beam was shone at the sample obliquely through the objective lens (CFI 60× Apochromatic TIRF, Nikon, Tokyo, Japan) for TIRF imaging. The donor and acceptor fluorescent signals were directed to the EMCCD (iXON Ultra897, Andor, Belfast, Northern Ireland, UK)) [74]. The experimental procedure and details are described as follows. First, the sample chamber was coated with 1 mg/mL biotinylated BSA and then 0.2 mg/mL NeutrAvidin. The sample solution was introduced to the chamber and incubated for the immobilization of the DNA tethers for 10 min. The chamber was then washed with a 4× chamber volume of buffer solution containing various concentrations of magnesium ions ([Mg^2+^]) and incubated for 30 min. Within 30 min, the B-Z transition normally reached equilibrium. For fluorescence measurements, the chamber was washed with oxygen scavenging solution (2 mM Trolox, 2.6 mM PCA (Protocatechuaic acid, Sigma-Aldrich, St. Louis, MO, USA), 0.4 unit/mL of rPCO (Protocatechuate 3,4-dioxygenase, OYC Japan, Tokyo, Japan)) supplemented with 5~500 mM MgCl_2_.

The experimental method is depicted schematically in Figure 4B. In our TIRF-based smFRET assay, the core in the B-state and Z-state exhibited the FRET efficiency (E_FRET_) of ~0.5 and ~0.15, respectively. In the assays to determine the degree of methylation or to compare the levels of methylation, we used [Mg^2+^] = 50 mM. The rationale for this choice is that at this concentration, cores with different degrees of methylation exhibit distinctive relative populations of B- and Z-states. In comparison with this reference information, we can determine the degree of methylation from the tested samples, measuring the activity of DMT and the inhibitory effect of dietary compounds on DMT.

## 4. Conclusions

Our assay allows us to quantitatively determine the degree of cytosine methylation. It employs DNA samples with a 22-bp CpG repeat sequence while traditional assays rely on a methylation event in a single CpG site, which is often subject to cleavage by methylation-sensitive nucleases. As a detection scheme, our method adopts the single-molecule FRET technique, which is sensitive enough to detect the structural transition of DNA at the level of a single molecule. We utilized the B-to-Z transition, which is promoted by cytosine methylation, and measured the propensity for the transition in a methylation-dependent manner. Thus, our assay is not affected by non-specific DNA cleavage or DNA degradation by a trace amount of unknown nuclease. Thanks to the high sensitivity of the single-molecule fluorescence technique, any multi-step amplification schemes based on nanoparticles or PCR are not required, as shown in a recent study [38]. By continuously measuring signals until dye photobleaching, kinetics information is obtainable by taking data for an extended length of time, which may carry distinctive signatures for underlying phenomena. By discerning different states of DNA methylation such as full- and hemi-methylation, this method can be optimized to study various types of substrates and methyltransferase enzymes which have diverse requirements for, and complex responses to, different substrates in DNA methylation. It is worth mentioning that our method is not designed for probing the methylation state of any (real and random) genomic loci or searching for sequences or genes with an altered methylation state, but for testing the activity of DMT enzymes and inhibitors because this method measures the degree of cytosine methylation based on the B-Z transition taking place in (dye-labeled) cytosine–guanine repeat sequences. Furthermore, the detection limit for DMT in this assay is rather high (~5~10 U/mL), higher than the values achievable by nuclease-dependent, amplification-based methods.

In summary, our DNA methylation assay simply detects the methylation-sensitive structural transition of individual DNA molecules, dispensing with nucleases and amplification steps. We envisage that it will allow us to study a broad spectrum of methylation-related problems.

## Data Availability

The data presented in this study are available on request from the corresponding author.

## Supplementary Materials

The supplementary material are available online at https://www.mdpi.com/article/10.3390/ijms222111990/s1.
