# Supplementary material for: Z-DNA as a Tool for Nuclease-Free DNA Methyltransferase Assay
Source: Int J Mol Sci. 2021 Nov 5;22(21):11990. doi: 10.3390/ijms222111990 (PMC8585049; doi:10.3390/ijms222111990)
Supplement: Supplementary file 1 [file ijms-22-11990-s001.zip › ijms-1425928-supplementary.pdf]

Supporting Information

Z-DNA as a tool for nuclease-free DNA methyltransferase assay

Sook Ho Kim[1,2,3,†], Hae Jun Jung[1,2,†], Seok-Cheol Hong[1,2,*]

[1]*Center for Molecular Spectroscopy and Dynamics, Institute for Basic Science, Seoul 02841, Korea.*

[2]*Department of Physics, Korea University, Seoul 02841, Korea.*

[3]*GRI-TPC International Research Center, Sejong University, Seoul 05006, Korea.*

Corresponding Author
*Seok-Cheol Hong
E-mail: hongsc@korea.ac.kr

Table of Contents

PREPARATION OF ENZYMATIC HEMI-METHYLATED (HM) MOLECULES 2
FIGURE S1 EFFECT OF [DMSO] ON DNA METHYLATION BY M.SSSI 3
FIGURE S2 Z-DNA FORMATION BY ENZYMATICALLY HEMI-METHYLATED DNA 4
FIGURE S3 E_{FRET} HISTOGRAMS OF QM AND TM CORES 5
FIGURE S4 METHYLATION TIME OF UM CORES BY M.SSSI 6
FIGURE S5 E_{FRET} HISTOGRAMS OF UM AND HM CORES TREATED WITH DMT 7
FIGURE S6 INEFFICIENT DE NOVO METHYLATION OF UM CORES BY DNMT1. 8
FIGURE S7 E_{FRET} HISTOGRAMS SHOWING DMT INHIBITION BY NATURAL DRUGS......10
FIGURE S8 DOI OF M.SSSI BY EGCG WITH LOW [SAM] 11
TABLE S1 SEQUENCE OF OLIGONUCLEOTIDES 12

Preparation of enzymatic hemi-methylated (HM) molecules.

First, we hybridize Cy5-labeled CG2 and unlabeled CG1 oligonucleotide strands and methylate cytosines within CG repeats in both strands with M.SssI. Then, it is heated to > 90°C, mixed with ~ 10 μM excess Cy3-labeled CG1 strand to outcompete with unlabeled CG1, and cooled down to room temperature. Now most of Cy5-labeled (methylated) CG2 is hybridized with Cy3-labeled CG1. In fact, Cy5-only Core molecules are not an issue because we only collect data from both Cy3- and Cy5-labeled DNA molecules. Full-methylated DNA molecules can be also obtained by direct methylation of FRET-pair-labeled duplex with M.SssI.

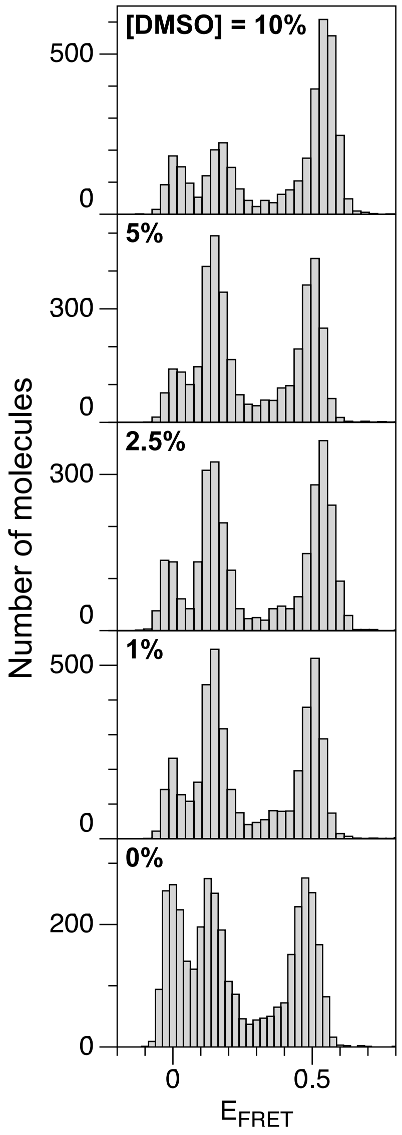

Figure S1 Effect of [DMSO] on DNA methylation by M.SssI.

For [DMSO] ≤ 5 %, the methylation activity of M.SssI remains unchanged. In the assay, we used [M.SssI] = 80 U/ml and performed FRET measurements in the presence of 50 mM Mg^{2+}.

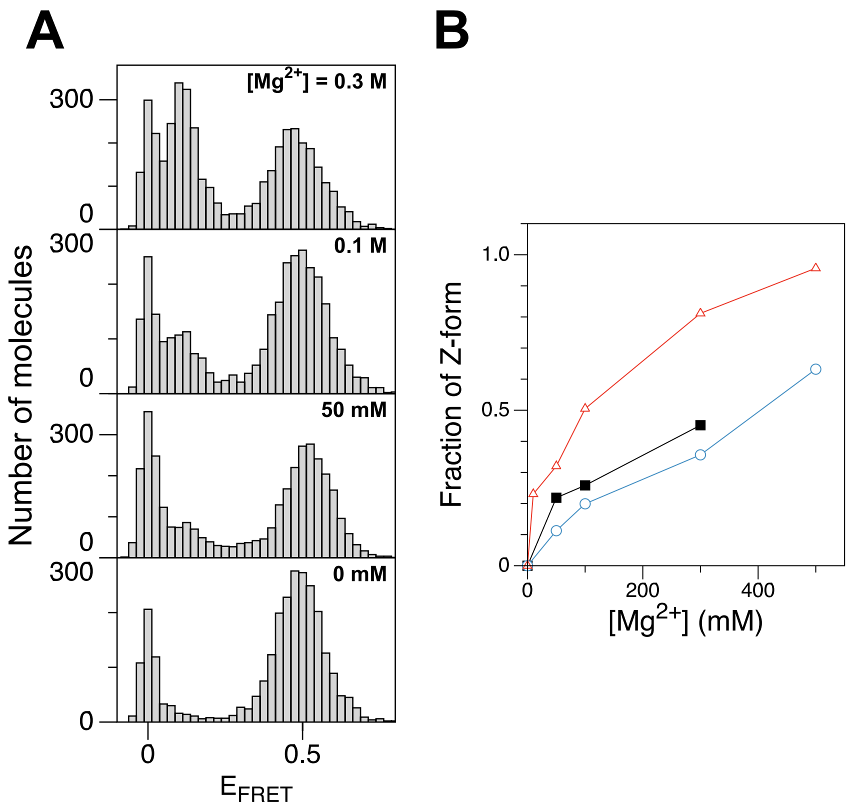

Figure S2 Z-DNA formation by enzymatically hemi-methylated DNA

(A) FRET efficiency histograms for the DNA Core 'hemi-methylated' in the enzymatic manner (see main and SI text; bottom to top: no Mg^{2+} (100 mM Na^{+} only), 50 mM, 100 mM, 300 mM Mg^{2+}).

(B) Fraction of Z-form vs. [Mg^{2+}] for DNA molecules with various conditions of cytosine methylation (black square: enzymatic hemi-methylation; blue circle: QM; red triangle: HM)

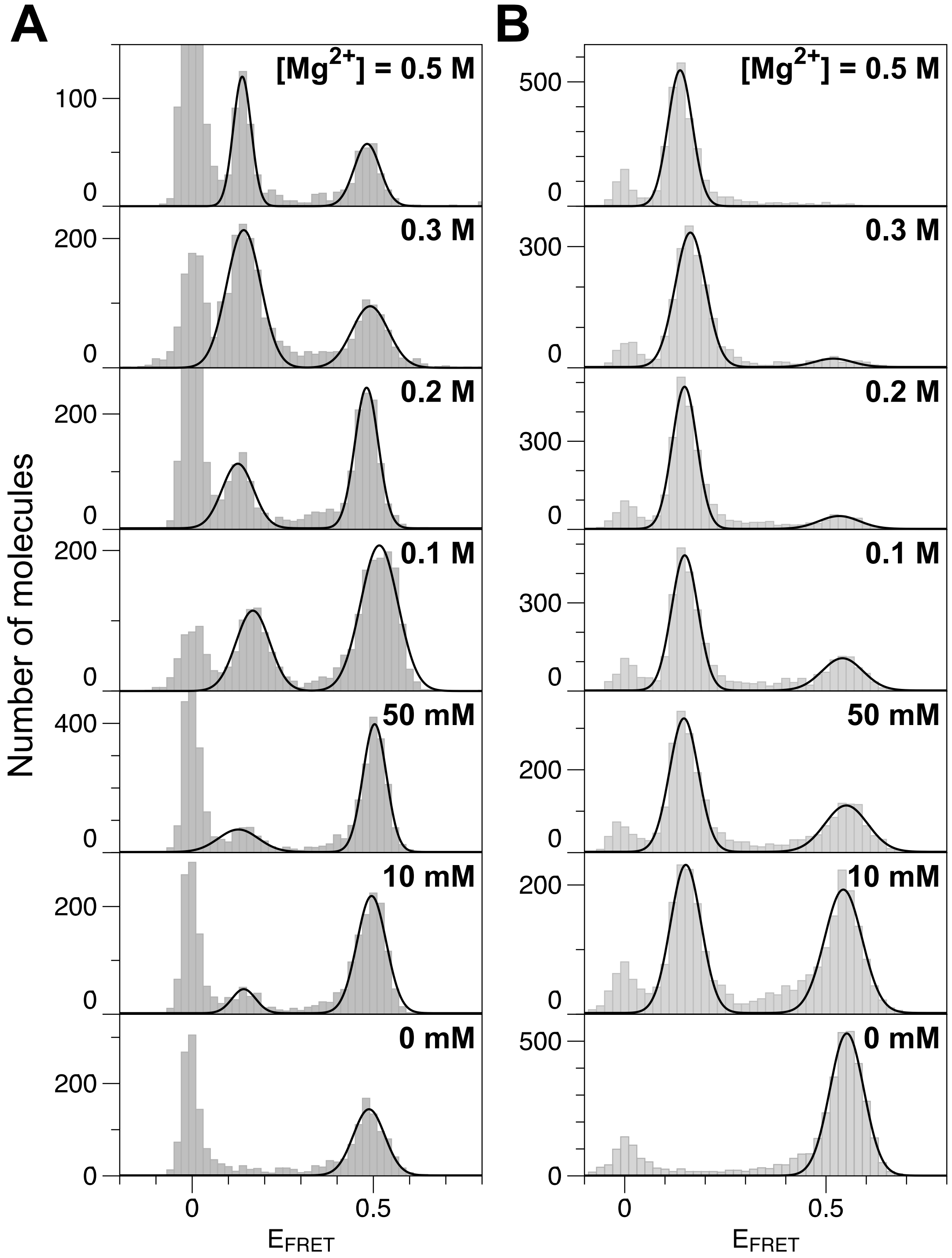

Figure S3 E_{FRET} histograms of QM and TM Cores.

(A) FRET efficiency histograms for a CG Core with 5 methylated cytosines on one strand (QM) (bottom to top: no Mg^{2+} (50 mM Na^+ only), 10 mM, 50 mM, 100 mM, 200 mM, 300 mM, 500 mM Mg^{2+})

(B) FRET efficiency histograms for a CG Core with 16 methylated cytosines (TM) (11 methylated cytosines on one strand and 5 methylated cytosines on the opposite strand) (bottom to top: no Mg^{2+} (50 mM Na^+ only), 10 mM, 50 mM, 100 mM, 200 mM, 300 mM, 500 mM Mg^{2+})

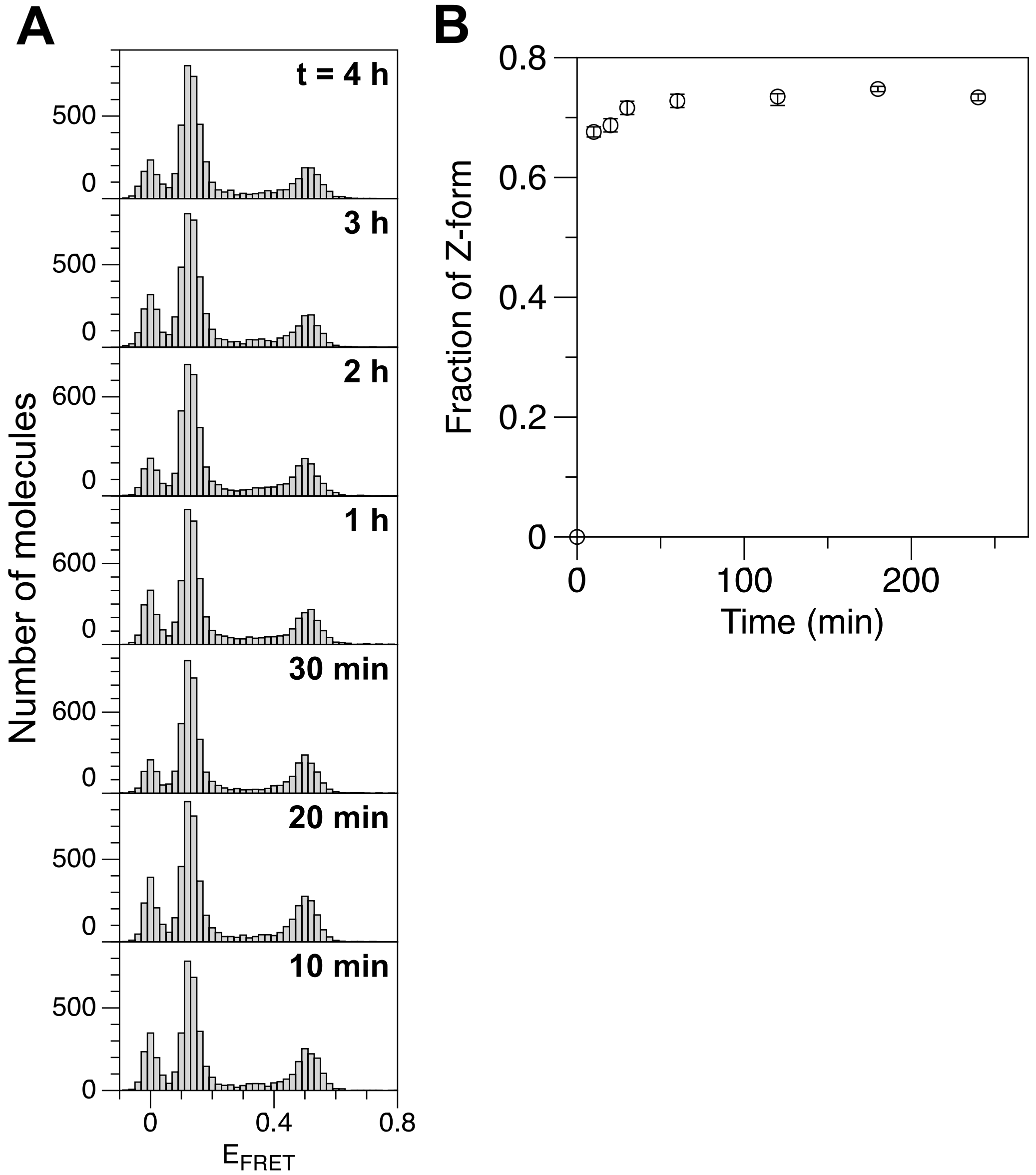

Figure S4 Methylation time of UM Cores by M.SssI

(A) FRET efficiency histograms from UM Cores for different methylation times by M.SssI (80 U/ml) in the presence of 50 mM Mg^{2+}.
(B) Fraction of Z-form as a function of methylation time by M.SssI.

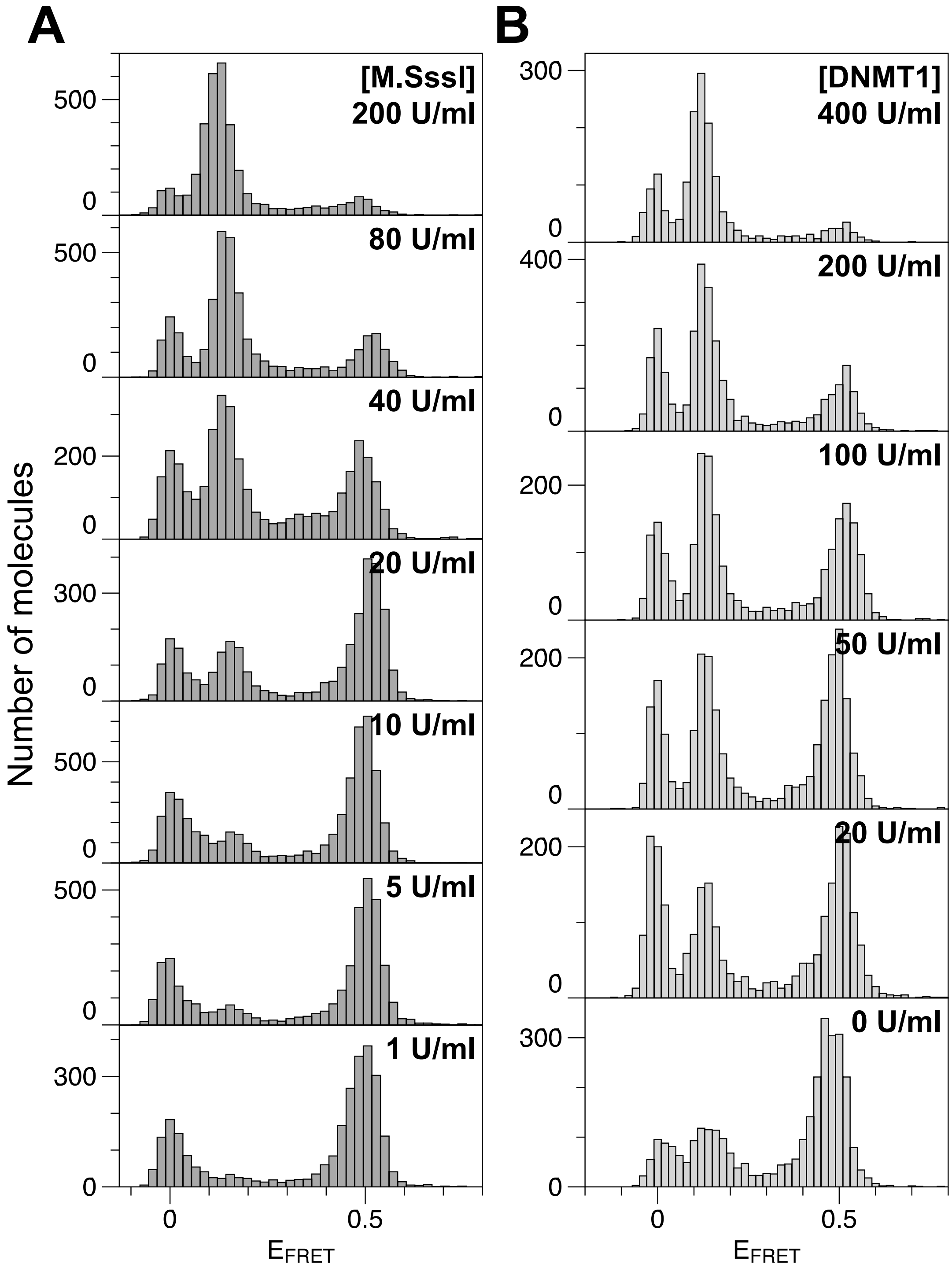

Figure S5 E_{FRET} histograms of UM and HM Cores treated with DMT

FRET efficiency histograms of initially UM or HM Cores after 2-hour incubation with various concentrations of M.SssI (1 – 200 U/ml) or DNMT1 (20 – 400 U/ml), respectively. Fractions of Z-form deduced therefrom are presented in Figure 3.

(A) FRET efficiency histograms from UM Cores after 2-hour incubation with M.SssI at the indicated concentrations (Figure 3A).

(B) FRET efficiency histograms from HM Cores after 2-hour incubation with DNMT1 at the indicated concentrations (Figure 3B). No-DNMT1 control from HM is shown at the bottom.

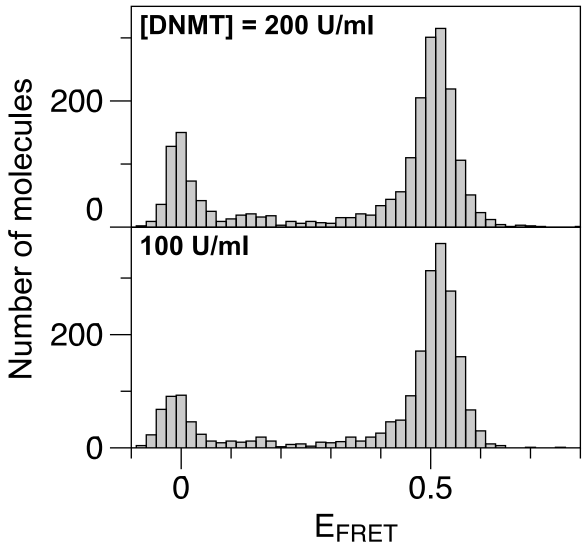

Figure S6 Inefficient *de novo* methylation of UM Cores by DNMT1.

FRET efficiency histograms from UM Cores pre-incubated with DNMT1 (100 or 200 U/ml) and in the presence of 50 mM Mg^{2+}.

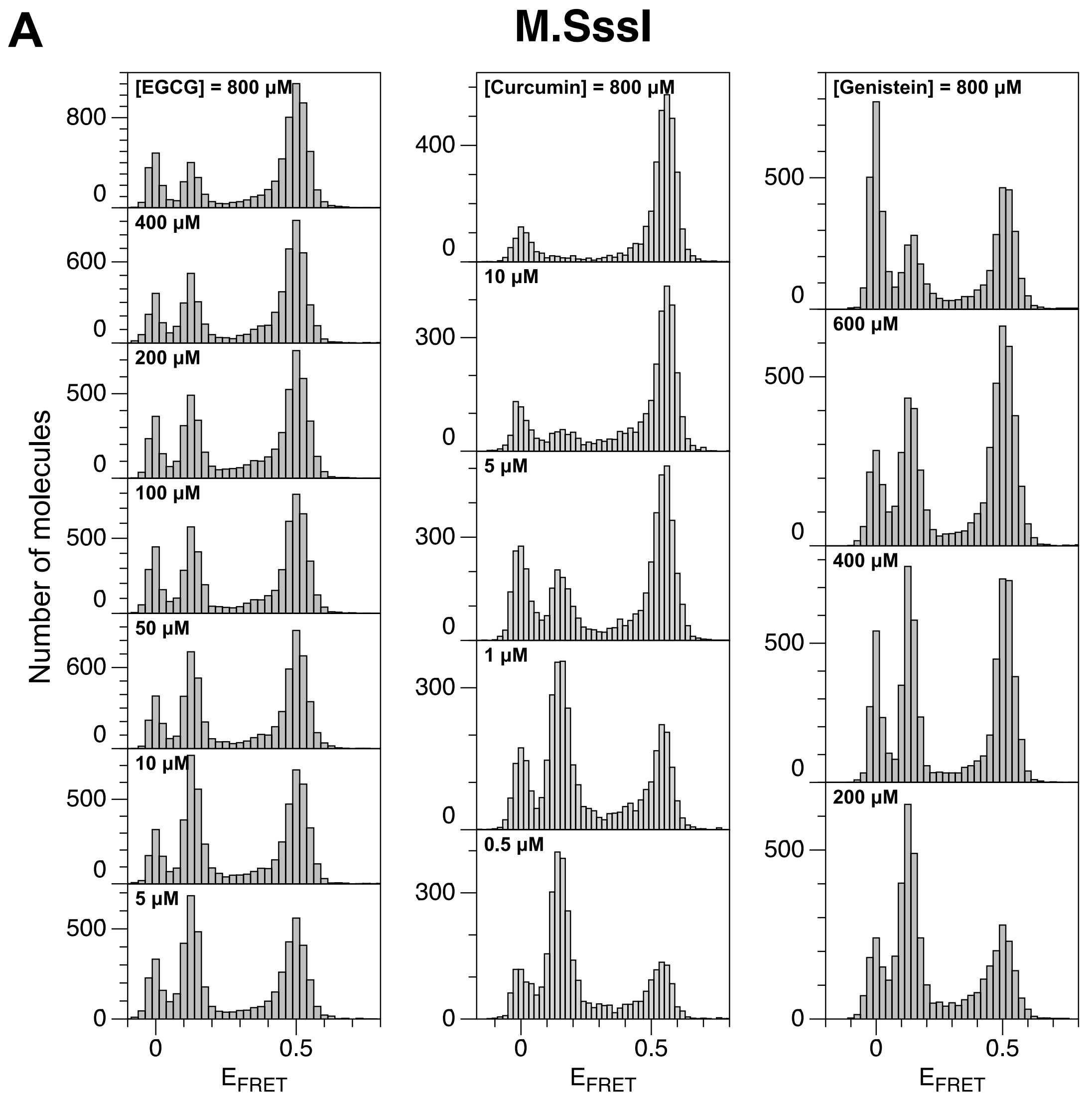
A
M.SssI
[EGCG] = 800 µM
400 µM
200 µM
100 µM
50 µM
10 µM
5 µM
[Curcumin] = 800 µM
10 µM
5 µM
1 µM
0.5 µM
[Genistein] = 800 µM
600 µM
400 µM
200 µM
Number of molecules
EFRET

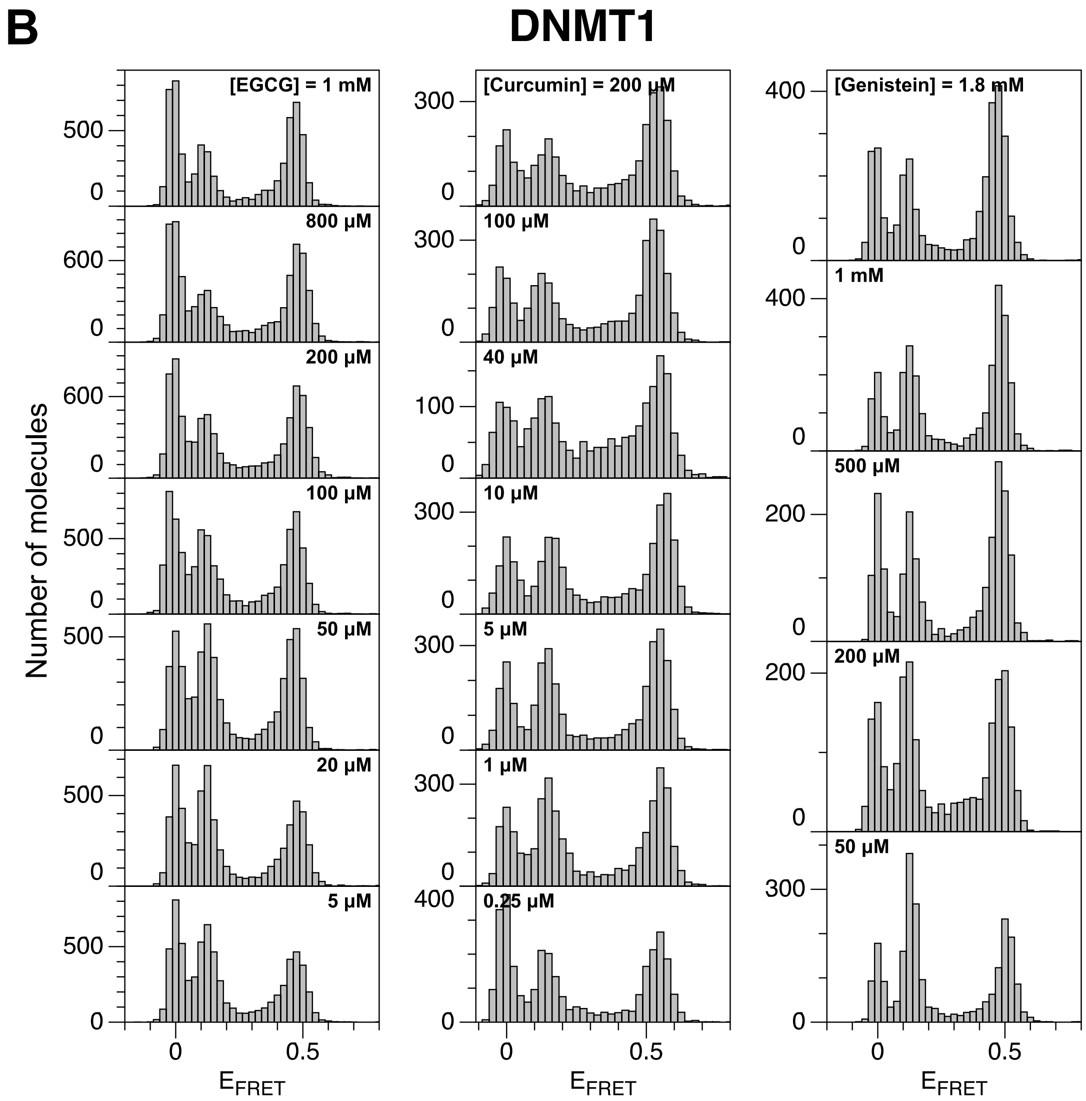

Figure S7 E_{FRET} histograms showing DMT inhibition by natural drugs.

(A) FRET efficiency histograms showing the inhibitory effects of the indicated natural compounds on the methylation activity of M.SssI.

(B) FRET efficiency histograms showing the inhibitory effects of the indicated natural compounds on the methylation activity of DNMT1.

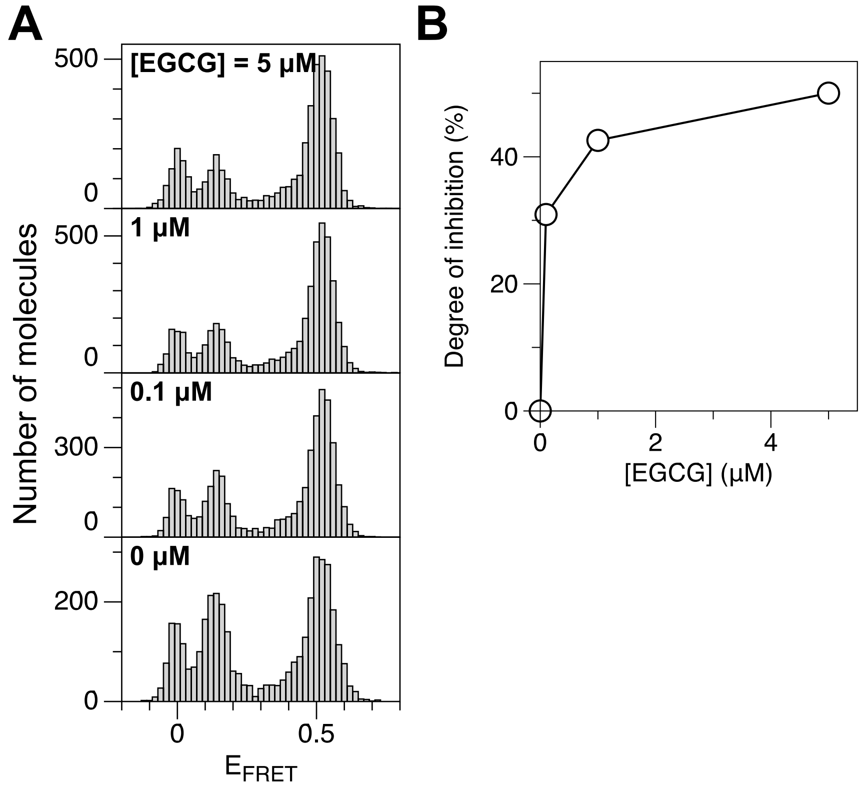

Figure S8 DOI of M.SssI by EGCG with low [SAM]

(A) FRET efficiency histograms showing the inhibitory effects of EGCG at various concentrations (0, 0.1, 1, and 5 μM) on the methylation activity of M.SssI in the presence of [SAM] = 10 μM.

(B) Degree of inhibition of M.SssI activity by EGCG from (A). The IC_{50} value of EGCG in cytosine methylation by M.SssI is significantly reduced to less than 5 μM with the reduced SAM concentration. This IC_{50} value of EGCG is in good agreement with reported IC_{50} values of EGCG obtained from similar experimental conditions (e.g. [SAM] = 10 – 20 μM).

Table S1 Sequence of oligonucleotides

Sequences of oligonucleotides used in this study. Each Core is constructed by hybridizing a pair of oligonucleotides (UM: CG1-CG2; QM: m^{5}CG1-CG2; HM: m^{11}CG1-CG2; TM: m^{5}CG1-m^{11}CG2; FM: m^{11}CG1-m^{11}CG2). UM, QM, and HM Cores are tethered by themselves by a biotin at 3' end of CG2(Bio) while TM and FM Cores are attached to the substrate via a biotin-labeled PCR fragment ligated to each Core. The PCR fragment is biotinylated by a PCR reaction containing biotin-dUTP, which is followed by BamHI digestion. Here, the sequence of scrambled DNA Core is also shown. iCy3 and iCy5 indicate internally labeled Cy3 and Cy5 dyes, respectively. 5′-Phos indicates the phosphate group at 5′ end for ligation. The Core molecules used in this study are annotated as follows: bold, Core sequence; blue, cohesive overhang; underlined C, methylcytosine.

Name	Sequence
CG1	5'-Phos-GAT CCT GGT A**CG CGC GCG CGC GCG CGC G**(iCy5)**CG CG**A TCG AC-3'
CG2	5'-GTC GAT **CGC GCG CGC GCG CGC GCG** (iCy3)**CGC G**TA CCA GGA TC(Bio)-3'
m^{5}CG1	5'-Phos-GAT CCT GGT A**CG CGC GCG CGC GCG CGC G**(iCy5)**CG CG**A TCG AC-3'
m^{11}CG1	5'-Phos-GAT CCT GGT A**CG CGC GCG CGC GCG CGC G**(iCy5)**CG CG**A TCG AC-3'
m^{11}CG2	5'-Phos-TGC CGT CGA T**CG CGC GCG CGC GCG CGC G**(iCy3)**CG CG**T ACC AG-3'
Scramble1	5'-Phos-GAT CCT GGT AGC TGC TCA GAC TCG TCA G(iCy5)CA TCA TCG AC-3'
Scramble2	5'-Phos-TGC CGT CGA TGA TGC TGA CGA GTC TGA G(iCy3)CA GCT ACC AG-3'
